# The temporal association between suicide and comorbid mental disorders in people treated for substance use disorders: a National registry study

**DOI:** 10.1186/s13722-023-00415-9

**Published:** 2023-10-11

**Authors:** Martin Ø. Myhre, Fredrik A. Walby, Jørgen G. Bramness, Lars Mehlum

**Affiliations:** 1 National Centre for Suicide Research and Prevention, Institute for Clinical Medicine, University of Oslo, Sognsvannveien 21, 0372 Oslo, Norway; 2 Department of Alcohol, Tobacco and Drugs, Norwegian Institute of Public Health, Oslo, Norway; 3 UiT– The Arctic University of Norway, Tromsø, Norway; 4 Norwegian National Competency Centre for Drug Abuse and Mental Illness, Brumunddal, Norway

**Keywords:** Suicide, Substance use disorders, Comorbid mental disorders, SUD services, Mental health services

## Abstract

**Background:**

The time after contact with specialized health services for mental health and substance use is associated with an increased risk of suicide, where temporal aspects of suicide and comorbid mental disorders in patients with substance use disorders could be associated. This study aimed to examine the temporal association between time from last treatment contact to suicide and comorbid mental disorders in patients with substance use disorders.

**Methods:**

This study is a historical prospective case series using nationwide registry data. It included 946 individuals registered the year before suicide with a substance use disorder (F10-F19) in Norway's specialized health services for treating substance use and mental health disorders between 2010 and 2020. The outcome was the number of weeks from the last contact with services to suicide. The exposure was comorbid mental disorders divided into 'no comorbid mental disorder’; ‘psychosis or bipolar disorders’ (F20−F31), ‘depressive or anxiety disorders' (F32−F49); and 'personality disorders' (F60-F69). Covariates included gender, age, last diagnosed substance use disorder, registered deliberate self-harm last year, and the number of in- and outpatient contacts the previous year.

**Results:**

The number of weeks from last service contact to suicide differed (*p* =  < 0.001) between patients with no comorbid mental disorders (Median = 7; IQR 2–23), psychosis or bipolar disorders (Median = 2; IQR = 1–7), depressive or anxiety disorders (Median = 3; IQR = 1–11) and personality disorders (Median = 1; IQR = 1–5.5). Significantly decreased adjusted incidence rate ratios (aIRR) were found for psychosis or bipolar disorders [aIRR = 0.67 (95% CI 0.53–0.85)] and personality disorders [aIRR = 0.56 (0.42–0.77)] compared to no comorbid mental disorder when adjusted for individual characteristics and service contact. For depressive and anxiety disorders compared to no comorbid mental disorder, the association was significant when adjusted for individual characteristics [aIRR = 0.55 (0.46–0.66)].

**Conclusions:**

While patients with substance use disorders generally died by suicide a short time after contact with services, patients with comorbid mental disorders died an even shorter time after such contact and significantly shorter than patients without such comorbidities.

**Supplementary Information:**

The online version contains supplementary material available at 10.1186/s13722-023-00415-9.

## Introduction

Individuals with substance use disorders (SUDs) have an increased risk of premature death [1]. SUDs include both alcohol use disorders (AUD) and drug use disorders (DUD), and both are associated with an increased risk of suicide [2]. A period where suicide risk peaks is the time after discharge from inpatient mental health services [3]. Whereas many studies have examined suicide in the wake of contact with psychiatric services, there is a shortage of studies on suicide risk after contact with SUD services. A study of patients in DUD treatment showed that suicide risk was highest during hospitalization and the first period after discharge [4]. Better knowledge of what factors influence this temporal aspect of suicide after contact with SUD services or mental health services could reduce the suicide risk in this high-risk group.

Studies have shown that comorbid schizophrenia, bipolar disorder, depression, and personality disorder double the suicide risk in SUD patients compared to patients without comorbidities [5–7]. Comorbid mental disorders are generally associated with increased service utilization in SUD patients [8]. Moreover, depression is regarded as a central risk factor for suicide in SUD patients [9], but other comorbid mental disorders are less described. This highlights the need to study comorbid mental disorders in SUD patients and their association with suicide in a broader manner. No standard categorization of comorbid mental disorders currently exists for SUD patients and suicidal behavior, and different studies have employed different categorizations. Previous studies have found an increased risk of suicide for past-year psychiatric treatment in DUD patients [10], involuntary admission to psychiatric services, and a diagnosis of affective disorder in SUD patients [11]. The additional risk of comorbid mental disorders also seems to be associated with temporal aspects of suicide after service contact, with studies finding that SUD patients frequently have outpatient contacts during the last year before their suicide [12] or, for patients with AUD or DUD in specialized health care, within two months before suicide [13, 14]. Moreover, adjusting for comorbid psychiatric disorders reduced differences in service contact within two weeks and within two months before death by suicide [13], indicating that increased service use was associated with comorbidities. When examining the use of specialized services during the last year, suicide in SUD patients with brief contact trajectories, consisting of few contacts distributed throughout the last year before suicide, was associated with having less comorbid mental disorders [15]. The suicides occurred longer after the last contact when compared with patients with denser service contact trajectories. These studies found associations between comorbid mental disorders and service use in the last year in SUD patients who died by suicide. They did not, however, explicitly examine the association between time from the last contact and comorbid mental disorders. When analyzing this temporal association, it is necessary to adjust for previous service use, as there may exist differences in previous service use between the groups that could affect temporal associations.

This study, therefore, aimed to examine the temporal association between time from last contact with services for substance use and mental health to suicide and comorbid mental disorders in SUD patients adjusted for individual characteristics and the intensity of service contact. To diminish the methodological challenge of the association between past service use and time from the last contact to suicide, it is necessary to adjust the association for service contact. We hypothesize that, in patients with comorbid psychosis or bipolar disorder, depressive or anxiety disorder, or personality disorders, suicide would occur a shorter time after the last contact with services compared with SUD patients without such comorbid mental disorders.

## Methods

### Data sources

The study adopted a historical prospective case series design based on a nationwide registry linkage of data from the Norwegian Cause of Death Registry (NCDR) [16] and the Norwegian Patient Registry (NPR) [17] using an 11-digit personal identifier, making it possible to follow individuals in different registries over time. The NPR conducted the linkage. The NCDR contains information about the causes of death for all deaths in Norway. The national coverage is very high (> 98%) [18], and 88% of suicides have been confirmed following reclassification [19]. The NPR includes information about contacts with secondary health services in Norway. The NPR contains person-identifiable data for mental health services from 2008 and for SUD services from 2009. The completeness of valid personal IDs in the NPR is > 99% from 2010 and onwards for publicly funded mental health and SUD services [20].

In Norway, SUD services are organized as separate interdisciplinary specialized health care services that constitute an entire treatment chain for SUDs and comorbid mental disorders, except severe mental disorders, such as psychosis disorders, which are treated in mental health services. Most specialized SUD treatment units in Norway are publicly funded, and their data are included in the NPR.

### Sample

First, information on all suicides in Norway between 2010 and 2020 based on ICD-10 codes (X60-X84; Y10-14; Y870; Y872) [21] was retrieved from the NCDR. These data on suicide cases were then linked to data from the NPR, through which suicide cases registered with contact with SUD or mental health services were identified during the last year before their death. This procedure identified 3,036 patients who had died by suicide and had had contact with services for substance use or mental health during the year before their death. Lastly, we obtained all patients with an ICD-10 diagnosis of SUD (F10-F16; F18-F19), which amounted to 946 patients.

### Variables

The variable of interest in this study was the number of weeks from the last contact with services for mental health or substance use to suicide. The variable was established by subtracting the date of the last contact with services from the NPR from the date of death from the NCDR, which gave the number of days, which subsequently was transformed into weeks. In the absence of a standard categorization of the exposure, we based the categorization of comorbid mental disorders on the clinical literature, separating between severe mental disorders or ‘dual diagnosis’ [22], episodic emotional and adjustment disorders, and personality disorders. The information on the exposure variable, comorbid mental disorders, was retrieved from registry data from the last service contact registered with a non-SUD-related ICD-10 psychiatric diagnosis (F20-F69), where we recorded the last diagnosis within the last year. We collapsed these diagnoses into three broad categories: ‘psychosis or bipolar disorder’ (F20-F29, F30-F31), ‘depressive or anxiety disorder’ (F32-F48), and ‘personality disorder’ (F60-F69).

Data on the date of death, method of suicide, gender, and age were retrieved from the NCDR. Method of suicide was collapsed into three categories: ‘hanging or strangulation’ (X70), ‘poisoning’ (X60-X67), and ‘other methods’ (X68-X69, X71-X82, Y10-Y34, and Y870, Y87.2). Age was used as a continuous variable. ICD-10 diagnoses for SUDs (F10-F16; F18-F19), level of care (in- or outpatient), and sector of contact (mental health or SUD services) at last contact were retrieved from the NPR. The number of contacts was measured by counting the number of in- or outpatient contacts with services for SUD and mental health in the last year. Inpatient suicide was measured as patients with a date of death before or equal to the date of last contact and who were discharged from services as dead. We categorized SUD as ‘alcohol’ (F10), ‘opiates’ (F11), ‘cannabinoids’ (F12), ‘sedatives and hypnotics’ (F13), ‘other substances’ (F14-F16) and ‘multiple substances’ (F19) using the last registered ICD-10 diagnosis of an SUD within the last year before the suicide. Deliberate self-harm was measured using the ICD-10 code X6n from emergency care episodes in the somatic datasets in the NPR from which the patients were discharged from health service contacts alive. The Charlson Comorbidity Index [23] was estimated using data from the NPR to examine somatic comorbidity, using it as an ordinal variable with levels 0, 1–2, and 3 or more.

### Analysis

Descriptive analyses of the distribution of covariates between comorbid mental disorders were performed using Chi-squared tests. For the outcome variable, we plotted the cumulative weekly percentage of suicides per week by comorbid mental disorders, estimated the median and interquartile range, and tested the overall difference in distribution between the groups using the Kruskal–Wallis non-parametric test.

The association between comorbid mental disorders and the number of weeks from the last contact to suicide was examined using count data regression modeling, with Poisson and negative binomial regression models. Individuals who died during an inpatient admission were excluded from the models. The model with the best fit, measured by the Akaike Information Criterion (AIC), was used. Models were checked for seasonality (calendar month) and trend (calendar year) by adding them as covariates in the models. They were controlled for in the analysis if coefficients were significant at a 5% level to increase model fit. Models were adjusted stepwise for variables that theoretically could confound the association—first, for individual characteristics (Model 1), and second, for individual characteristics and the number of inpatient admissions and outpatient contacts (Model 2). Service use was planned to be included as a continuous variable, but as the variables were heavily skewed to the left, we used them as categorical variables. We conducted a sensitivity analysis on the service contact variables, comparing two variables (number of inpatient admissions and number of outpatient contacts) with four variables (number of inpatient admissions and number of outpatient contacts in SUD services and mental health services, respectively). The fit of the final models was compared using AIC and likelihood ratio tests. To check the robustness of the association, we examined whether the sector or level of care at last contact moderated the associations in Models 1 and 2 in post-hoc analyses.

The hypothesis and analysis plan were preregistered at the Open Science Framework (10.17605/OSF.IO/5USC2), and the data were analyzed using R version 4.1.2 [24]. Ggplot2 [25] was used to build the figure, and the MASS package [26] was used for the negative binomial regression models. The analysis code is available at the Open Science Framework (10.17605/OSF.IO/3ZT5J).

## Results

Of all 946 individuals within the study period 2010–2020 who had died from suicide within one year after their last contact with SUD services, 170 individuals (17.8%) had received a diagnosis of psychosis or bipolar disorder, 338 (35.7%) had received a diagnosis of depressive or anxiety disorder, and 81 (8.6%) had received a diagnosis of personality disorder, whereas 357 individuals (37.7%) had not received any comorbid mental disorders. Further characteristics of the sample are provided in Table 1. The occurrence of undetermined codes (Y10-Y34) for the underlying cause of death was very low (*n* = 15; 0.01%). Of the patients in contact with services for SUD or mental health during the last year before suicide, 59 individuals (6.2%) died during an inpatient admission. These individuals were excluded from the subsequent analysis.

**Table 1:** Study population

	No comorbidity *n* = 357	Psychosis/bipolar disorder*n* = 170	Depression/anxiety *n* = 338	Personality disorder *n* = 81	Overall *n* = 946	*p*
Male gender *n* (*%*)	275 (77.0)	114 (67.0)	211 (62.4)	32 (39.5)	632 (66.8)	** < 0.001**^1^
Age median (1st-3rd quartile*)*	41 (30, 51)	37 (29, 47)	44 (31, 55)	37 (25, 45)	40 (30, 51)	** < 0.001**^2^
Suicide method *n (%)*						**0.002**^1^
Hanging/strangulation	167 (46.8)	76 (44.7)	140 (41.4)	35 (43.2)	418 (44.2)	
Poisoning	95 (26.6)	37 (21.8)	111 (32.8)	35 (43.2)	278 (29.4)	
Other means	95 (26.6)	57 (33.5)	87 (25.7)	11 (13.6)	250 (26.4)	
Last substance use disorder						** < 0.001**^1^
Alcohol (F10)	153 (42.9)	50 (29.4)	157 (46.4)	33 (40.7)	393 (41.5)	
Opiates (F11)	84 (23.5)	13 (7.6)	30 (8.9)	8 (9.9)	135 (14.3)	
Cannabinoids (F12)	28 (7.8)	20 (11.8)	29 (8.6)	8 (9.9)	85 (9.0)	
Sedatives and hypnotics (F13)	20 (5.6)	11 (6.5)	48 (14.2)	6 (7.4)	85 (9.0)	
Other substances (F14-F16)	21 (5.9)	18 (10.6)	19 (5.6)	5 (6.2)	63 (6.7)	
Multiple substances (F19)	51 (14.3)	58 (34.1)	55 (16.3)	21 (25.9)	185 (19.6)	
Deliberate self-harm last year	17 (4.8)	15 (8.8)	57 (16.9)	22 (27.2)	111 (11.7)	** < 0.001**^1^
Charlson comorbidity index						0.137^1^
0	234 (65.5)	125 (73.5)	214 (63.3)	57 (70.4)	630 (66.6)	
1–2	112 (31.4)	45 (26.5)	116 (34.3)	22 (17.2)	295 (31.2)	
> 2	11 (3.1)	< 3	8 (2.4)	< 3	21 (2.2)	
Service at last contact						** < 0.001**^1^
SUD services	253 (70.9)	24 (14.1)	111 (32.8)	25 (30.9)	413 (43.7)	
Mental health services	104 (29.1)	146 (85.9)	227 (67.2)	56 (69.1)	533 (56.3)	
Level of care at last contact						**0.013**^1^
Inpatient	125 (35.0)	75 (44.1)	134 (39.6)	43 (53.1)	377 (39.9)	
Outpatient	232 (65.0)	95 (55.9)	204 (60.4)	38 (46.9)	569 (60.1)	
Inpatient admissions last year						** < 0.001**
0	150 (42.0)	18 (10.6)	47 (13.9)	12 (14.8)	227 (24.0)	
1	140 (39.2)	40 (23.5)	105 (31.1)	13 (16.0)	298 (31.5)	
2-	67 (18.8)	112 (65.9)	186 (55.0)	56 (69.1)	421 (44.5)	
Outpatient contacts last year						** < 0.001**
0	56 (15.7)	11 (6.5)	30 (8.9)	3 (3.7)	100 (10.6)	
1–10	219 (61.3)	61 (35.9)	154 (45.6)	31 (38.3)	465 (49.2)	
11-	82 (23.0)	98 (57.6)	154 (45.6)	47 (58.0)	381 (40.3)	

Description of SUD patients who died by suicide within one year after contact with services for the treatment of substance use and mental health disorders by comorbid mental disorders

^1^Chi-squared test

^2^Mann-Whitney test

**Fig. 1:**
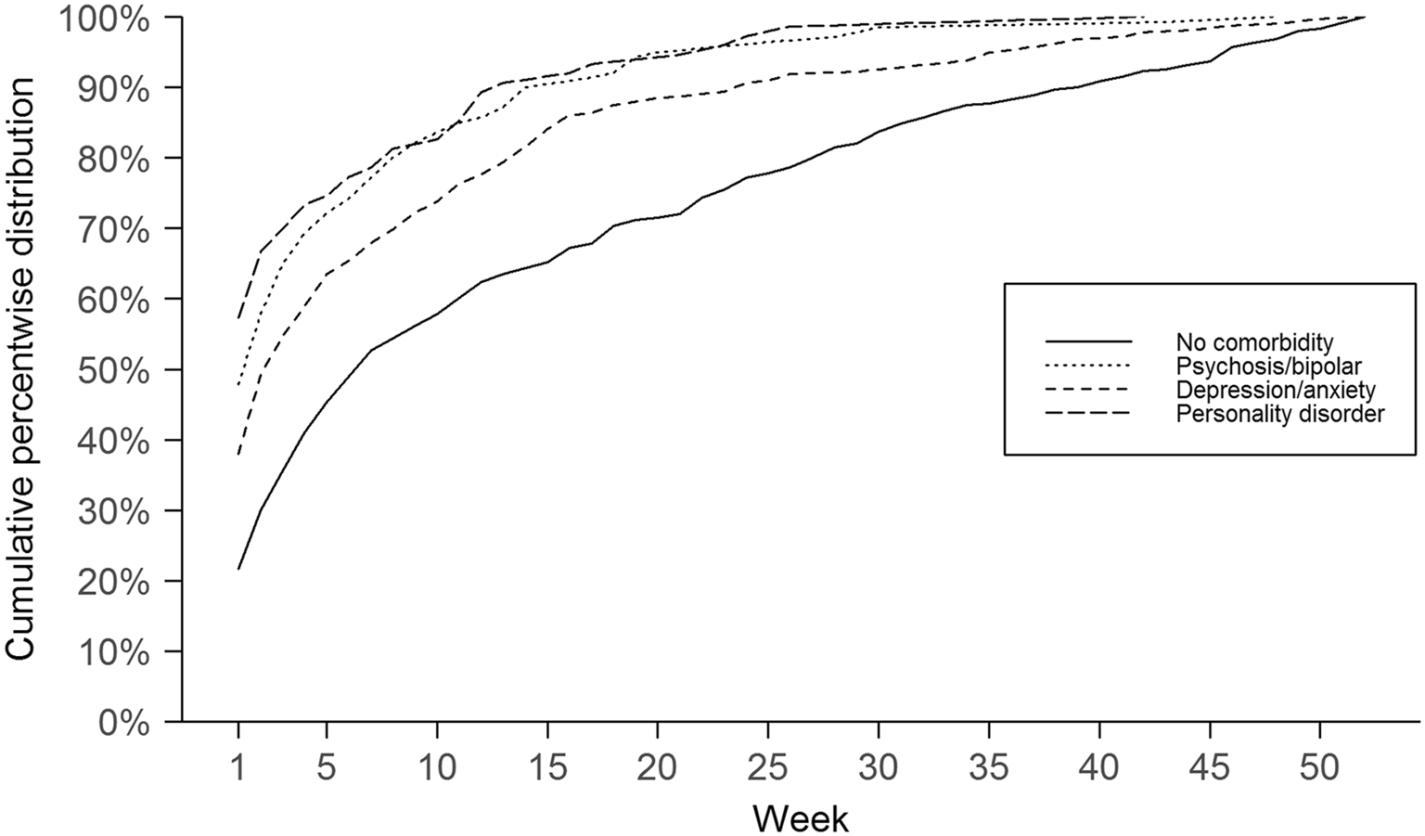
Cumulative percentwise incidence of the number of suicides by week from the last contact with services for substance use and mental health. Comorbid mental disorders are illustrated by the lines in the figure

The median number of weeks from the last contact to suicide was 7 (IQR = 2–23) for no comorbid mental disorder, 2 (IQR = 1–7) for psychosis or bipolar disorder, 3 (IQR = 1–11) for depressive and anxiety disorders, and 1 (IQR = 1–5.5) for personality disorders, with an overall significant difference between the groups (*p* =  < 0.001). For all groups, the percentwise incidence of suicide was highest in the first weeks after contact, with patients with comorbid mental disorders showing consistently higher incidence in the first weeks compared with patients without comorbid mental disorders (Fig. 1). Among suicides occurring during the first weeks after contact, a diagnosis of personality disorder was most frequent, followed by psychosis and bipolar disorder. The depression or anxiety disorder group had a lower weekly incidence in the first weeks after contact than the psychosis or bipolar and personality disorders but followed a similar increase in cumulative incidence as the psychosis or bipolar and personality disorders.

In general, the negative binomial models had a better fit than the Poisson models, and the former models were therefore used. In the bivariate model (Model 1), as shown in Table 2, psychotic or bipolar disorder (IRR = 0.38 (95% CI 0.31–0.49), *p* < 0.001), depressive or anxiety disorders (IRR = 0.57 (0.48–0-68), *p* < 0.001), and personality disorders (IRR = 0.32 (0.24–0.43), *p* < 0.001) were all significantly associated with reduced incidence rate ratios compared to the no comorbidity group, indicating that suicides occur after fewer weeks after contact with services in these groups. In Model 2, the association between time from last contact to suicide and having comorbid mental disorders compared to no comorbid mental disorder remained significant (*p* =  < 0.001 for all coefficients) with minor changes in the IRRs when controlling for gender, age, type of SUD, and deliberate-self-harm (psychosis or bipolar disorder IRR = 0.39 (0.31–0.49); depressive or anxiety disorder IRR = 0.55 (0.46–0.66); personality disorders IRR = 0.35 (0.26–0.49)). In Model 3, controlling for both individual characteristics from Model 2 and the intensity of service contact measured by the number of in- and outpatient contacts the last year, being registered with either a comorbid psychosis/bipolar disorder (IRR = 0.67 (0.53–0.85), *p* =  < 0.001) or personality disorder (IRR = 0.56 (0.42–0.77), *p* =  < 0.001) were significant while being registered with a depressive/anxiety disorder was not significant (IRR = 0.86 (0.72–1-03), *p* = 0.106). We found no significant differences when we stratified the models by the level of care and the type of service at the last contact (available in Additional file 1).

**Table 2:** The temporal association between suicide and comorbid mental disorders

Variable	Model 1		Model 2		Model 3	
	IRR (95% CI)	*p*	IRR (95% CI)	*p*	IRR (95% CI)	*p*
Comorbid mental disorders						
No comorbidity	1 (Ref)		1 (Ref)		1 (Ref)	
Psychosis/bipolar disorder (F20-F31)	0.38 (0.31–0.49)	** < 0.001**	0.39 (0.31–0.49)	** < 0.001**	0.67 (0.53–0.85)	** < 0.001**
Depressive/anxiety disorder (F32-F49)	0.57 (0.48–0.68)	** < 0.001**	0.55 (0.46–0.66)	** < 0.001**	0.86 (0.72–1.03)	0.106
Personality disorders (F60-F69)	0.32 (0.24–0.43)	** < 0.001**	0.35 (0.26–0.49)	** < 0.001**	0.56 (0.42–0.77)	** < 0.001**
Gender						
Male			1 (Ref)		1 (Ref)	
Female			0.89 (0.75–1.05)	0.165	0.96 (0.0.82–1.12)	0.583
Age			1.01 (1.01–1.02)	** < 0.001**	1.01 (1.00–1.01)	0.083
Substance use disorder						
Alcohol related disorders (F10)			1 (Ref)		1 (Ref)	
Opiate related disorders (F11)			0.87 (0.69–1.10)	0.237	1.00 (0.81–1.25)	0.988
Cannabis related disorders (F12)			1.02 (0.76–1.39)	0.898	1.05 (0.80–1.39)	0.745
Sedative, hypnotic, or anxiolytic related disorders (F13)			1.36 (1.03–1.82)	**0.032**	1.35 (1.04–1.78)	**0.023**
Other substance related disorders (F14-F16)			1.03 (0.75–1.44)	0.858	1.03 (0.76–1.41)	0.855
Multiple substance related disorders (F19)			1.08 (0.86–1.37)	0.491	1.11 (0.89–1.39)	0.317
Deliberate self-harm						
No deliberate self-harm last year			1 (Ref)		1 (Ref)	
Deliberate self-harm last year			0.78 (0.61–1.01)	**0.049**	0.94 (0.74–1.20)	0.593
Number of admissions						
0					1 (Ref)	
1					0.74 (0.61–0.91)	**0.002**
≥ 2					0.37 (0.37–0-56)	** < 0.001**
Number of outpatient contacts						
0					1 (Ref)	
1–10					0.64 (0.50–0.82)	** < 0.001**
≥ 11					0.28 (0.22–0.37)	** < 0.001**

Model 1 = Adjusted for seasonality. Model 2 = Model 1 adjusted for gender, age, substance use disorder and deliberate self-harm. Model 3 = Model 2 adjusted for number of in- and outpatient contacts in SUD and mental health services. Significant differences (*p* < 0.05) are marked with bold text

Negative binomial regression models of the association between number of weeks from the last contact and comorbid mental disorders in SUD patients who died by suicide within one year after contact with SUD or mental health services

The fit of Model 2 (*x*^*2*^ = 30.145, *p* = 0.001) and Model 3 (*x*^*2*^ = 288.077, *p* =  < 0.001) was better than of Model 1, with Model 3 having the best overall fit (Model 2 AIC = 5753.662; Model 3 AIC = 5553.586). The sensitivity analysis indicated that the fit of Model 3 was better with the number of inpatient admissions and outpatient contacts collapsed into two variables (AIC = 5581.06), compared to inpatient admissions and outpatient contacts separated for SUD and mental health services (AIC = 5593.06) (see Additional file 2).

## Discussion

Our main finding was that all types of comorbid mental disorders were associated with a shorter time from the last contact to suicide than patients without such comorbidities. This association was retained when adjusting for gender, age, type of SUD, and having a diagnosis of deliberate self-harm in the last year before suicide. In the final model, where we even adjusted for the intensity of service contact through the number of in- and outpatient contacts over the last year, we found that the association was attenuated but still retained for comorbid psychosis, bipolar disorders, and personality disorders, and reduced to non-significant for depressive and anxiety disorders. Accordingly, we found considerable support for our hypothesis that SUD patients with comorbid mental disorders end their lives by suicide within a significantly shorter time after their last contact with services than SUD patients with no such comorbidity.

In Model 2, we adjusted for gender, age, deliberate self-harm, and type of SUD, which affected the estimates minimally. Sedative, hypnotic, and anxiolytic use disorders, associated with more weeks to suicide compared to AUD, were the only associations retained when adjusting for past-year service contact. The increased time from the last contact to suicide in sedative, hypnotic and anxiolytic use disorders indicates a potential temporal subgroup. The SUD variable we used here does not capture other aspects of SUDs, such as severity or relapse, which could be associated with temporal aspects. However, SUD severity and relapse could be indirectly associated with increased service use, which we adjusted for in the subsequent Model 3. Deliberate self-harm showed a limited association with the temporal association, decreasing the number of weeks from the last contact to suicide compared to no deliberate self-harm. However, the estimate attenuated after adjusting for past year service use, which could indicate that it is associated with service contact—an aspect that future studies should examine further.

Adjusting for the number of in- and outpatient contacts during the last year in Model 3 attenuated the estimates more than only adjusting for individual characteristics in Model 2. Service contact reportedly tends to increase before suicide in SUD patients [13, 14]. As baseline levels of service contact may differ between comorbid mental disorders, it is important to control for these differences to avoid reverse causation when the time from last service contact to suicide is under study in association with comorbid mental disorders. In our models, the attenuation observed was similar across all groups of comorbid mental disorders, and the non-significant association for depressive and anxiety disorders after adjustment seems to be due to a weaker association before adjusting for service use and not because of more attenuation. Another possibility for the differences observed in the models is that the models could be under-adjusted, and the estimates for psychosis or bipolar disorders or personality disorders also would also be reduced to non-significant with further adjustment.

It is also important to consider exceptions to the temporal association in our study. Previously, we have described an intermittent contact trajectory weakly associated with comorbid mental disorders but with a similar temporal distribution of time from contact to suicide as trajectories more strongly associated with comorbid mental disorders [15]. Levola and colleagues [11] found a significant association between unipolar and bipolar depression and suicide, but not psychosis, anxiety, adjustment, and personality disorders after inpatient psychiatric care. An important point to consider regarding this exception is that the study was of a cohort from one treatment center and only examined inpatient psychiatric care, whereas, in our study, we included a broader range of specialized services and a national sample.

The risk of suicide is high in SUD patients with comorbid mental disorders such as schizophrenia [22] and personality disorders [23]. Moreover, have similar hazard ratios been found for all mental disorders in SUD patients, except personality disorders, which had higher hazard ratios [5]. Here, the temporal association is less pronounced and less robust for depressive or anxiety disorders. It is plausible that the depressive or anxiety disorders group is more heterogeneous than the other groups and could thus contain more variation in suicide risk. Nevertheless, this points to a broader set of comorbid mental disorders that are associated with temporal aspects of suicide in SUD patients after contact with services for SUD and mental health.

### Implications for clinical care

This study describes the period of highly increased suicide risk after contact with services – the post-contact period [3]. Given the current lack of effective suicide preventive methods for patients with SUDs, more detailed knowledge of patients' clinical features during this high-risk period might be a necessary basis for developing novel preventive approaches [2, 27]. While this study cannot describe the risk or effectiveness of treatment, it can inform policies and guide suicide prevention efforts that should be subject to further examination.

Since prediction of suicide risk is challenging even in clinical populations [28], policies or practices to prevent suicide must target more than individuals with identified high risk. Thus, scalable policies and practices that can be implemented widely are needed to affect the suicide rates in clinical populations [27]. Based on these findings, preventive policies aimed at the period immediately after contact, such as follow-up after discharge or safety planning, could benefit patients with severe mental disorders or personality disorders. In contrast, strategies aimed at long-term follow-up, such as lowering the readmission threshold, could benefit patients without comorbid mental disorders. Overall, it shows the necessity of a range of preventive strategies implemented in parallel to sustain patient safety after service contact.

In addition to the broader policies and practices mentioned above, the strong temporal association could suggest that comorbid mental disorders should be targets for clinical interventions to reduce suicide risk in this heterogeneous group. While evidence about suicide preventive interventions targeting SUD patients is currently sparse [27], reduced suicide rates have been found in services that implemented a dual diagnosis policy [29], which indicates a potential for prevention that requires further examination. Additionally, given the close temporal association between service contact and suicide, it is suggested that patient safety measures should be implemented more systematically simultaneously with treatment. Examples could include brief interventions that improve crisis management and facilitate help-seeking behavior, such as s*afety planning* [30], or service-related features, such as increasing the availability of crisis services [29].

### Strengths and limitations

This study used preregistration of its hypotheses and analysis plan and utilized national data, which minimizes selection bias and provides a comparatively large study. Another strength is that we have controlled the temporal association for past-year service use, thereby reducing problems with reverse causation. The study also included outpatient contacts, which are less examined. We conducted post-hoc stratified analyses separating whether the last contact was as in- or outpatient and found no difference. Furthermore, we included all specialized services in both SUD and mental health services, which was necessary as SUD patients who die by suicide often have contact with both types of specialized services [11].

Our study is a historical prospective case series and includes only individuals who died by suicide. Moreover, the study included only deaths with recorded undetermined or suicidal intent in the NCDR. Underreporting of suicides is likely to be present, as under-recording of drug intoxications as suicides have been found [31]. We based the recording of inpatient suicides on whether the patient was being discharged as dead in the NPR, which may lead to some inpatient suicides being misclassified as discharged due to some underreporting. The overall magnitude is, however, most likely minor and not considered to have affected the model significantly. Moreover, some underreporting is expected since we used clinical (not structured interview-based) ICD-10 diagnoses from the NPR to assess comorbid mental disorders. Previous studies have found underreporting to be a more pronounced problem for depression than for severe mental disorders, where the correspondence between diagnoses from the registries and structured diagnostic assessment is higher [32, 33]. This study used clinical diagnoses recorded in the registries, making a more fine-grained assessment of comorbid mental disorders difficult, hence supporting a crude categorization. Lastly, the NPR only includes specialized SUD and mental health services, not including primary care, where SUDs are also treated. Moreover, treatment rates for AUD are generally low [34], indicating that SUDs are undertreated, affecting generalizability to the entire population.

## Conclusions

While patients with substance use disorders in general died by suicide a short time after contact with services, patients with comorbid mental disorders died an even shorter time after such contact, and significantly shorter than patients without such comorbidities. For patients with comorbid psychosis, bipolar, or personality disorders, this association was present even when adjusting for service use. This indicates that the association tends to be independent of service use. Increased understanding of the factors affecting the temporal distribution of suicide post-contact has implications for developing suicide prevention policies and the design of health services for patients with SUDs.

### Supplementary Information

**Additional file 1: **Post-hoc analysis of the association between weeks from the last contact and comorbid mental disorders by sector and level of care at last contact.

**Additional file 2: **AICs log likelihood tests between of the Poisson and Negative binomial regression models.

## Data Availability

The data used in this study is indirectly identifiable and used under license from the Norwegian Institute of Public Health and The Norwegian Directorate of Health. Due to the licenses, access to the data is restricted and cannot be shared openly to protect study participant privacy. Data are located in controlled access data storage at the University of Oslo. The analysis code is openly available from the Open Science Framework (10.17605/OSF.IO/3ZT5J).

## Abbreviations

AIC: Akaike information criteria
AUD: Alcohol use disorder
DUD: Drug use disorder
IRR: Incidence rate ratio
NCDR: Norwegian cause of death registry
NPR: Norwegian patient registry
SUD: Substance use disorder
