# Supplementary material for: The temporal association between suicide and comorbid mental disorders in people treated for substance use disorders: a National registry study
Source: Addict Sci Clin Pract. 2023 Oct 11;18:59. doi: 10.1186/s13722-023-00415-9 (PMC10568834; doi:10.1186/s13722-023-00415-9)
Supplement: Supplementary file 1 — Additional file 1: Post-hoc analysis of the association between weeks from the last contact and comorbid mental disorders by sector and level of care at last contact. [file 13722_2023_415_MOESM1_ESM.pdf]

Additional file 1. Post-hoc analysis of the association between weeks from the last contact and comorbid mental disorders by sector and level of care at last contact.

	Model 1		Model 2	
	IRR (95 % CI)	*p*	IRR (95 % CI)	*p*
Sector at last contact				
SUD services				
No comorbidity	1 (ref)		1 (ref)	
Psychosis or bipolar disorder	0.26 (0.16-0.45)	**< 0.001**	0.27 (0.16-0.47)	**< 0.001**
Depressive or anxiety disorder	0.61 (0.48-0.80)	**< 0.001**	0.63 (0.48-0.82)	**< 0.001**
Personality disorders	0.37 (0.23-0.62)	**< 0.001**	0.44 (0.26-0.76)	**0.001**
Mental health services				
No comorbidity	1 (ref)		1 (ref)	
Psychosis or bipolar disorder	0.35 (0.26-0.49)	**< 0.001**	0.35 (0.25-0.48)	**< 0.001**
Depressive or anxiety disorder	0.49 (0.37-0.65)	**< 0.001**	0.47 (0.36-0.63)	**< 0.001**
Personality disorders	0.25 (0.17-0.39)	**< 0.001**	0.28 (0.18-0.44)	**< 0.001**
Level of care at last contact				
Inpatient				
No comorbidity	1 (ref)		1 (ref)	
Psychosis or bipolar disorder	0.40 (0.27-0.61)	**< 0.001**	0.38 (0.24-0.58)	**< 0.001**
Depressive or anxiety disorder	0.56 (0.42-0.75)	**< 0.001**	0.55 (0.41-0.75)	**< 0.001**
Personality disorders	0.33 (0.22-0.52)	**< 0.001**	0.36 (0.22-0.59)	**< 0.001**
Outpatient				
No comorbidity	1 (ref)		1 (ref)	
Psychosis or bipolar disorder	0.39 (0.29-0.52)	**< 0.001**	0.38 (0.29-0.51)	**< 0.001**
Depressive or anxiety disorder	0.60 (0.48-0.75)	**< 0.001**	0.57 (0.45-0.72)	**< 0.001**
Personality disorders	0.27 (0.18-0.41)	**< 0.001**	0.28 (0.18-0.45)	**< 0.001**

Note. Model 1 adjusted for seasonality. Model 1 adjusted for seasonality, gender, age, substance use disorder and deliberate self-harm
