# Supplementary material for: The temporal association between suicide and comorbid mental disorders in people treated for substance use disorders: a National registry study
Source: Addict Sci Clin Pract. 2023 Oct 11;18:59. doi: 10.1186/s13722-023-00415-9 (PMC10568834; doi:10.1186/s13722-023-00415-9)
Supplement: Supplementary file 2 — Additional file 2: AICs log likelihood tests between of the Poisson and Negative binomial regression models. [file 13722_2023_415_MOESM2_ESM.pdf]

Additional file 2. AICs log likelihood tests between of the Poisson and Negative binomial regression models

	Poisson regression		Negative binomal regression				
Model (adjustments)	df	AIC	df	AIC	Log lik	x^2	*p*
Model 1 (seasonality)	15	13250.451	16	5762.291	-2865.2		
Model 2 (seasonality, gender, age, SUD, deliberate self-harm)	23	12887.42	24	5749.574	-2850.8	28.717	< 0.001
Model 3 (seasonality, gender, age, SUD, deliberate self-harm, inpatient contacts, outpatient contacts)	27	11071.702	28	5579.166	-2761.6	178.408	< 0.001
